# Potential role of 4-hydroxyisoleucine in enhancing fertility in male mice with diet-induced obesity

**DOI:** 10.3389/fendo.2025.1561543

**Published:** 2025-08-21

**Authors:** Emmanuel Osei Nkansah, Yunzhu Lan, Hui Zhang, Binbin Xu, Qiaodan Li, Mohammad Ishraq Zafar, Jian Xu

**Affiliations:** Department of Obstetrics and Gynecology, Center for Reproductive Medicine, The Fourth Affiliated Hospital of School of Medicine and International School of Medicine, International Institutes of Medicine, Zhejiang University, Yiwu, Zhejiang, China

**Keywords:** obesity, 4-hydroxyisoleucine, 4-HIL, male fertility, metabolic disorder

## Abstract

**Background:**

Obesity is associated with hormonal imbalance, increased oxidative stress, and inflammation in the testis. These conditions adversely affect sperm quality, leading to impaired male fertility. Therefore, therapeutic interventions to counteract the adverse effects of obesity are crucial. This study explored the therapeutic effects of 4-hydroxyisoleucine (4-HIL) on fertility in male mice with diet-induced obesity.

**Methods:**

C57BL6 male mice (n=45) were randomly divided into normal diet (ND, n=15) and high-fat diet (HFD, n=30) groups for 10 weeks. The HFD group was then randomized into untreated (HFD, n=15) and 4-HIL-treated (200 mg/kg/day, intraperitoneal injection, HFD + 4-HIL group, n=15) for 6 weeks. ND and HFD controls received saline (0.3 mL/30 g body weight) throughout the intervention period. Comprehensive evaluations included (1) metabolic assessments (body weight, glucose, insulin and pyruvate tolerance tests, and blood serum lipids), (2) sperm analysis (count, concentration, and morphology), (3) fertility testing (mating trials and *in vitro* fertilization), (4) testicular histopathology (fat deposition and apoptosis), (5) biochemical assays (reproductive hormones, oxidative stress markers, and inflammatory cytokines), and (6) molecular analyses (mRNA sequencing and qPCR validation of differentially expressed genes).

**Results:**

4-HIL treatment improved metabolic parameters, including reduced weight gain, enhanced glucose tolerance, and optimized blood serum lipids, compared to HFD controls. Treated mice exhibited superior sperm quality with increased count and concentration, reduced histomorphological abnormalities in the testis, and attenuated oxidative stress and inflammation. Furthermore, the key spermatogenic gene expressions, including spem1 and spata24, were significantly optimized in the testes of mice treated with 4-HIL compared to those of untreated mice (HFD group).

**Conclusion:**

This study demonstrates that 4-HIL therapy ameliorates obesity-induced testicular dysfunction and improves fertility markers in mice. The beneficial effects of this compound on metabolic parameters, sperm quality, and spermatogenic gene expression suggest its potential as a therapeutic agent for obesity-related male infertility. Further research is warranted to elucidate the underlying mechanisms and assess the clinical translatability of these findings.

## 1 Introduction

Obesity is an adverse health condition characterized by excessive accumulation of adipose tissues. Its high incidence is largely due to unhealthy dietary habits, including intake of hypercaloric diets and reduced physical activity (1, 2). Obesity is linked to increased oxidative stress and a high systemic inflammatory state in tissues, leading to chronic health conditions such as type II diabetes (3), cancer (4, 5), and compromised fertility (6–8).

Obesity decreases sperm production in humans and mice (9–11) by disrupting the hypothalamic-pituitary-gonadal (HPG) axis, testicular steroidogenesis, and testicular metabolic regulation (12, 13), thereby compromising fertility. Testosterone, a key hormone that regulates spermatogenesis and secondary sexual characteristics in male mammals, is reduced in men with obesity (14, 15) and severe inflammatory conditions (16). Oxidative stress markers, including malondialdehyde (MDA) and superoxide dismutase (SOD), have been used to demonstrate high oxidative stress in the testes of mice with obesity (13). Obesity causes lipid accumulation in the testes and disrupts testicular thermoregulation, negatively affecting germ cell differentiation (17).

Given the debilitating effects of obesity, the search for therapeutic interventions to restore normal body weight and mitigate the resulting adverse conditions, including obesity-induced male subfertility, is crucial. 4-Hydroxyisoleucine (4-HIL) is a naturally occurring amino acid derivative found in fenugreek seeds (*Trigonella foenum-graecum*). This bioactive component has garnered significant attention for its potential therapeutic benefits, particularly in managing metabolic disorders such as obesity and diabetes (18, 19). Studies have shown that 4-HIL promotes weight loss, reduces fatty liver, and optimizes lipid profiles in the context of obesity, while also improving insulin sensitivity and lowering blood glucose levels in diabetes (20–24). These metabolic benefits suggest that 4-HIL has broader applications for improving overall metabolic health.

Although no study has specifically investigated the effects of 4-HIL on male fertility, its known benefits for metabolic health suggest potential positive outcomes. This preliminary study aimed to determine whether 4-HIL enhances fertility in male mice with diet-induced obesity. We hypothesized that 4-HIL may act on several interconnected factors, specifically mitigating inflammation, oxidative stress, and metabolic dysregulation, all of which are well-established factors that negatively affect male reproductive function. By targeting these factors, 4-HIL could help restore a more favorable metabolic environment for improving fertility.

## 2 Materials and methods

### 2.1 Ethics

The Institutional Review Board of the Laboratory Animal Center of Zhejiang University (ethical approval number: ZJU20230462) reviewed and approved this study protocol. All experiments in this study were performed in accordance with the approved protocols and regulations of the Institutional Review Board of the Laboratory Animal Center, Zhejiang University. This study conformed to the ARRIVE guidelines (25). A checklist of the ARRIVE guidelines is provided in **Supplementary File 2**.

### 2.2 Mice and grouping

Forty-five C57BL/6 male mice (4 weeks old) were purchased from Charles River Laboratories China Inc. and housed under aseptic conditions with a sustained room temperature of 22°C and humidity (60% relative humidity), with 12:12 h light and dark cycles (light cycle 08:00 a.m. to 8.00 pm), at the Laboratory Animal Center of the International Institutes of Medicine, Zhejiang University. After the mice arrived at the center, they were allowed to acclimatize for one week on a normal control diet (D12450J; Research Diets Inc., New Brunswick, NJ, USA). Following acclimatization, the mice were randomly divided into two groups: normal control (ND) group (n=15) and high-fat diet (HFD) group (n=30). The ND group was fed a normal control diet, whereas the HFD group was fed a 60% high-fat diet (XTHF60%; Jiangsu Synergy Pharmaceutical Biological Engineering Co. Ltd., China) (26) for ten weeks. At the end of the 10^th^ week of HFD feeding, the HFD group was further divided into two groups: HFD group (n=15) and HFD + 4HIL group (n=15). All groups were maintained with dedicated feeding for the next six weeks during the planned intervention.

### 2.3 Intervention

ND Group: During treatment, the mice continued to be fed a normal diet daily per mouse and were not given any medications, except that they were injected with 0.3 ml/30 g saline for 6 weeks.
HFD Group: During treatment, the mice continuously fed with high fat-diet daily per mouse and were not given any medications, except they were injected with saline volume of 0.3 ml/30 g for 6 weeks.
HFD + 4-HIL Group: Mice were fed a high-fat diet daily and treated with 200 mg/kg/day intraperitoneal injections of 4-hydroxyisoleucine (Cas: 781658-23-9, Aladdin Scientific) in 0.3 ml saline per 30 g body weight for six weeks (19).

The body weights of the mice in all groups were measured and recorded weekly during and at the end of the intervention.

### 2.4 Sampling

At the end of the intervention, the mice were anesthetized with Avertin (280 mg/kg) (13) and sacrificed by cervical dislocation, and samples of interest were harvested. Blood sera were collected by centrifugation (3000 rpm, room temperature for 20 min). The blood sera collected were kept in a temperature condition of – 80°C in a refrigerator. The testes and epididymal adipose tissues were immediately retrieved, weighed, and stored at -80°C. Some of the retrieved testes were fixed in 4% paraformaldehyde for histological analysis and TUNEL assay, while others were stored in liquid nitrogen for Oil Red O staining, mRNA sequencing, qPCR, oxidative stress, and inflammatory status assessments.

### 2.5 Fertility test

Three male mice were randomly selected from each group and allowed to mate with three normal C57BL/6 female mice (8 weeks old), with one male mouse paired with a female mouse (1:1) in a cage until the females were found to be pregnant (confirmed by abdominal bulging) before isolation. The number of days until positive pregnancy and the number of offspring (male and female) in each group were recorded.

### 2.6 Glucose tolerance test (GTT)

Before GTT, five mice were randomly selected from each group and fasted for 6 h. Blood was collected from the tail vein of each mouse at 0 min, and the fasting blood glucose concentration was recorded using an ACCU-CHEK Active glucometer (Roche). A single dose of 2 g/kg intraperitoneal injection of glucose was administered to the mice, and glucose concentrations in the blood were recorded at 15, 30, 60, and 120 min.

### 2.7 Insulin tolerance test (ITT)

Before the ITT investigation, five mice were randomly selected from each group and fasted for 6 h. Blood samples were collected from the tail vein, and the fasting blood glucose concentration at 0 min was recorded using an ACCU-CHEK Active glucometer (Roche). Mice were then administered a single dose of 0.75IU/kg intraperitoneal injection of insulin aspart (300IU in 300 ml) placed in 0.2 ml saline (per mouse), after which glucose concentrations in blood were recorded at 15, 30, 60, and 120 min.

### 2.8 Pyruvate tolerance test (PTT)

Before the PTT investigation, five mice were randomly selected from each group and fasted for 6 h. Blood samples were collected from the tail vein and the fasting blood glucose concentration at 0 min was recorded using an ACCU-CHEK Active glucometer (Roche). Mice were then administered a single dose of 1.5 g/kg intraperitoneal injection of sodium 2-oxopropanoate (Cas: 113-24-6) dissolved in phosphate-buffered saline (PBS), and the glucose concentrations in the blood were recorded at 15, 30, 60, and 120 min.

### 2.9 Sperm count and concentration analysis

M199 culture solution (Cat. No: KGL1402-500, Jiangsu KeyGEN Bio TECH Corp., Ltd.) was placed in a centrifuge tube and preheated at 37°C. One side of the epididymis was placed in 0.6 mL of M199 culture solution for 1–2 min. The epididymis was cut with scissors and placed at 37 °C for 3–5 min. The sperm suspension was diluted in 1:1 ratio, and the sperm count was determined using a TOX IVOS sperm analyzer. Sperm suspension was placed in a Leja counting plate. The counting plate was then placed on the microscope stage, and the sperm movement image was captured using a microscope camera and video recorder with a 10× inverted objective lens.

### 2.10 Sperm morphological examination

A drop of the sperm suspension was placed approximately 1.5 cm from one end of each slide. The sperm droplets were spread along the edge of the slide to an appropriate width and pushed forward smoothly to create a sperm smear. Sperm smears were allowed to dry, fixed with methanol for at least 5 min, rinsed with water, stained using an automatic staining machine, sealed, and stored. Using Leica DM6000 B microscope, the position of sperm with less overlap was observed. Sperm morphology was assessed sequentially, and structurally intact spermatozoa were counted. Non-count cases included heads without tails, unclear outlines, and heads overlapping with other sperm. False double-head or tail sperm caused by sperm overlap were distinguished.

### 2.11 H&E staining

The testicular tissue was immersed in 4% paraformaldehyde and dehydrated using graded ethanol. The dehydrated testicular tissue was embedded in paraffin. Paraffin sections (4 μm thick) were obtained using a microtome (Leica, USA) and further processed with hematoxylin and eosin (H&E) following standard laboratory protocols. Finally, the images were observed under a light microscope at different magnifications, and alterations in testicular histology were captured.

### 2.12 Oil O Red staining

Frozen testes sections were thawed to room temperature, fixed for 15 min, and washed with distilled water. Oil Red O working solution was prepared, and testicular slices mounted on slides were immersed in the Oil Red O dye solution for 8–10 minutes (covered to avoid light). The slides were then immersed in two cylinders of 60% isopropyl alcohol for differentiation for 3 s and 5 s, respectively. The slides were then immersed in two tanks of distilled water for 10 s. The slides were dipped in hematoxylin for 3–5 minutes and soaked in three tanks of distilled water for 5, 10, and 30 s. The staining effect was assessed using light microscopy. Positive staining indicated that fat-laden cells appeared orange-red to bright red.

### 2.13 Tunel assay

Paraformaldehyde (4%)-fixed testes were embedded in paraffin. Paraffin-embedded sections were generated and dewaxed prior to staining. These sections were then covered with Protease K and incubated at 37 °C for 20 min. The sections were mounted on slides, placed in phosphate-buffered saline (PBS), washed, and dried. TDT enzyme was mixed with dUTP and added to cover the tissue, which was then placed in a wet box and incubated at 37°C for one hour. The sections were then washed three times with PBS (pH 7.4) for 5 min each. The PBS was then removed, and DAPI dye solution was added to the sections and incubated at room temperature for 10 min in the dark. Images were captured using fluorescence microscopy. The nuclei of normal testicular cells appeared blue (stained by DAPI), whereas apoptotic nuclei in the testes appeared red (from the TMR fluorescence TUNEL kit).

### 2.14 *In vitro* fertilization

Superovulation in female C57BL6 mice (eight weeks old) was achieved by intraperitoneal injection of pregnant mare serum gonadotropin (PMSG) at a dose of 10 IU, followed by a second intraperitoneal injection of human chorionic gonadotropin (hCG) at a dose of 10 IU (48 h later). Culture dishes for sperm capacitation, insemination and embryo growth were prepared and placed in an incubator with 5% CO_2_ at 37°C. Male mice (C57BL/6) were sacrificed, and their cauda epididymides were collected and placed in a sperm capacitation dish containing 150 μl of human tubal fluid (MR-070-D, Sigma-Aldrich, EmbryoMax^®^ HTF) covered with mineral oil to allow sperm capacitation. Cumulus-oocyte complexes (COCs) were then obtained from the oviductal ampulla of the superovulated mice 14 h after hCG injection and placed in an insemination dish containing a 150 μl drop of human tubal fluid (MR-070-D Sigma-Aldrich EmbryoMax^®^ HTF) covered with mineral oil. Finally, a 10 μl drop of the capacitated sperm suspension was placed in the human tubal fluid (MR-070-D Sigma-Aldrich EmbryoMax^®^ HTF) medium containing COCs. Subsequently, the sperm-COCs mixtures were incubated at 37°C in 5% CO_2_ for 6 h to generate zygotes. Zygotes were placed in an embryo growth dish containing 50 μl of KSOM medium (with BSA) covered with mineral oil. Images of zygotes were captured using a microscope. Two-cell embryos were observed 24 h after zygote formation. Four-cell embryos were assessed within 24 hours after two-cell embryos were observed. Eight-cell embryos and morulae were assessed at 24 h, and blastocysts at 48 h, after the formation of four-cell embryos.

### 2.15 Reproductive hormone assessment

The ELISA kits for testosterone (Cat No: BPE20375) with a detection range of 1–160 nmol/L, follicle-stimulating hormone (Cat No: BPE20419) with a detection range of 0.5–80 IU/ml, and luteinizing hormone (Cat No: BPE20343) with a detection range of 0.3–48 mIU/ml were obtained from Shanghai Lengton Bioscience Co., Ltd. Testosterone, follicle-stimulating hormone, and luteinizing hormone were measured using the aforementioned kits (following the manufacturer’s instructions), and the measurements were normalized.

### 2.16 Oxidative stress and inflammation assessment

The ELISA kits for MDA (Cat No: BPE20347) with a detection range of 0.15-28.8 nmol/ml, SOD (Cat No: BPE20348) with a detection range of 0.1–24 ng/ml, TNFα (Cat No: BPE20220) with a detection range of 4–640 ng/L, and IL-6 (Cat No: BPE20012) with a detection range of 2–320 ng/L were obtained from Shanghai Lengton Bioscience Co., Ltd. MDA concentration, SOD activity, TNF-α concentration, and IL-6 concentration were measured using the aforementioned kits (following the manufacturer’s instructions), and these measurements were normalized.

### 2.17 Serum lipid assessment

Serum lipid profiling kits, including low-density lipoprotein cholesterol (Cat No: A113-1-1), high-density lipoprotein cholesterol (Cat No: A112-1-1), and triglycerides (Cat No: A110-1-1), were obtained from the Nanjing Jiancheng Bioengineering Institute. The concentrations of low-density lipoprotein cholesterol, high-density lipoprotein cholesterol, and triglycerides were determined using the aforementioned kits (following the manufacturer’s instructions).

### 2.18 RNA isolation and library preparation

RNA was isolated using TRIzol reagent (Invitrogen, Carlsbad, CA, USA) according to the manufacturer’s protocol. RNA concentration and purity were determined using a NanoDrop ND-1000 (NanoDrop, Wilmington, DE, USA). The integrity of the retrieved RNA samples was assessed using a Bioanalyzer 2100 (Agilent, CA, USA), which was further validated by agarose gel electrophoresis. To purify poly (A) RNA from 1μg of total RNA, Dynabeads Oligo (dT) 25-61005 (Thermo Fisher Scientific, CA, USA) was used. The purified poly (A) RNA was then broken down using the Magnesium RNA Fragmentation Module (NEB, cat.e6150, USA) at 94°C for 5–7 min. Complementary DNA (cDNA) was generated using SuperScript II Reverse Transcriptase (Invitrogen, cat. no. 1896649, USA) and single- or dual-index adapters were generated and sliced into the fragments. AMPureXP beads were used to select the sliced fragments. The joined products were amplified using the following PCR conditions: denaturation at 95°C for 3 min, followed by 8 cycles of denaturation at 98°C for 15 s, annealing at 60°C for 15 s, extension at 72°C for 30 s, and extension at 72°C for 5 min. Illumina Novaseq™ 6000 was used to conduct 2×150bp paired-end sequencing (PE150) as per the protocol approved by LC-Biotechnology Co., Ltd., Hangzhou, Zhejiang, P.R. China.

### 2.19 Bioinformatics analysis of mRNA-sequencing

Bioinformatic analysis of mRNA sequencing was conducted using FASTP (https://github.com/OpenGene/fastp), HISAT2 (https://ccb.jhu.edu/software/hisat2), and gffcompare (https://github.com/gpertea/gffcompare/). mRNA expression levels were determined using StringTie. Fold change > 2 or < 0.5, and parametric F-test with R package edgeR (https://bioconductor.org/packages/release/bioc/html/edgeR.html) (p > 0.05) were used to estimate differentially expressed mRNAs.

### 2.20 Quantitative PCR

TRIzol (Invitrogen, 15596026) was used to isolate total RNA from the testis homogenates, according to the manufacturer’s protocol. *TransScript* ^®^ All-in-One First-Strand cDNA Synthesis SuperMix (Cat. No. AT341) was used to synthesize cDNA. qPCR was performed using Green qPCR SuperMix (*PerfectStart* ^®^, Cat. No. AQ602), and the target gene expression levels were optimized for β-actin expression and calculated based on the comparative cycle threshold method (2^−△△Cq^). Three biological replicates were used in all experiments. The source of all primers listed in **Table 1** is PrimerBank

**Table 1:** Primer sequences used for the analysis of mRNA expression levels of spem1 and spata24.

*spem1*	F	GGGTGGGCCTCGTATCAAAAC
*spem1*	R	ATGCGGATTTGGCTCCAGAG
*spata24*	F	TACACGGGCTCTTTACCAACA
*spata24*	R	GACTGGACTTGCCTGTATCCT
*Beta actin*	F	GGCTGTATTCCCCTCCATCG
*Beta actin*	R	CCAGTTGGTAACAATGCCATGT

### 2.21 Statistical analysis

Data were analyzed using GraphPad Prism v. 8. To assess the statistical significance of the differences between the study group variables, we used the Student’s t-test (for pairwise comparison between the two groups) and ANOVA (to compare means across three or more groups). The results of these statistical tests were considered statistically significant at the following thresholds: * p < 0.05 (indicating significant), ** p < 0.01 (moderate significance), *** p< 0.001 (high significance), and **** p < 0.0001 (extremely high significance).

## 3 Results

### 3.1 4-HIL therapy reduces excess adipose tissue, insulin resistance, and dyslipidemia in male mice with diet induced obesity

**Figure 1:**
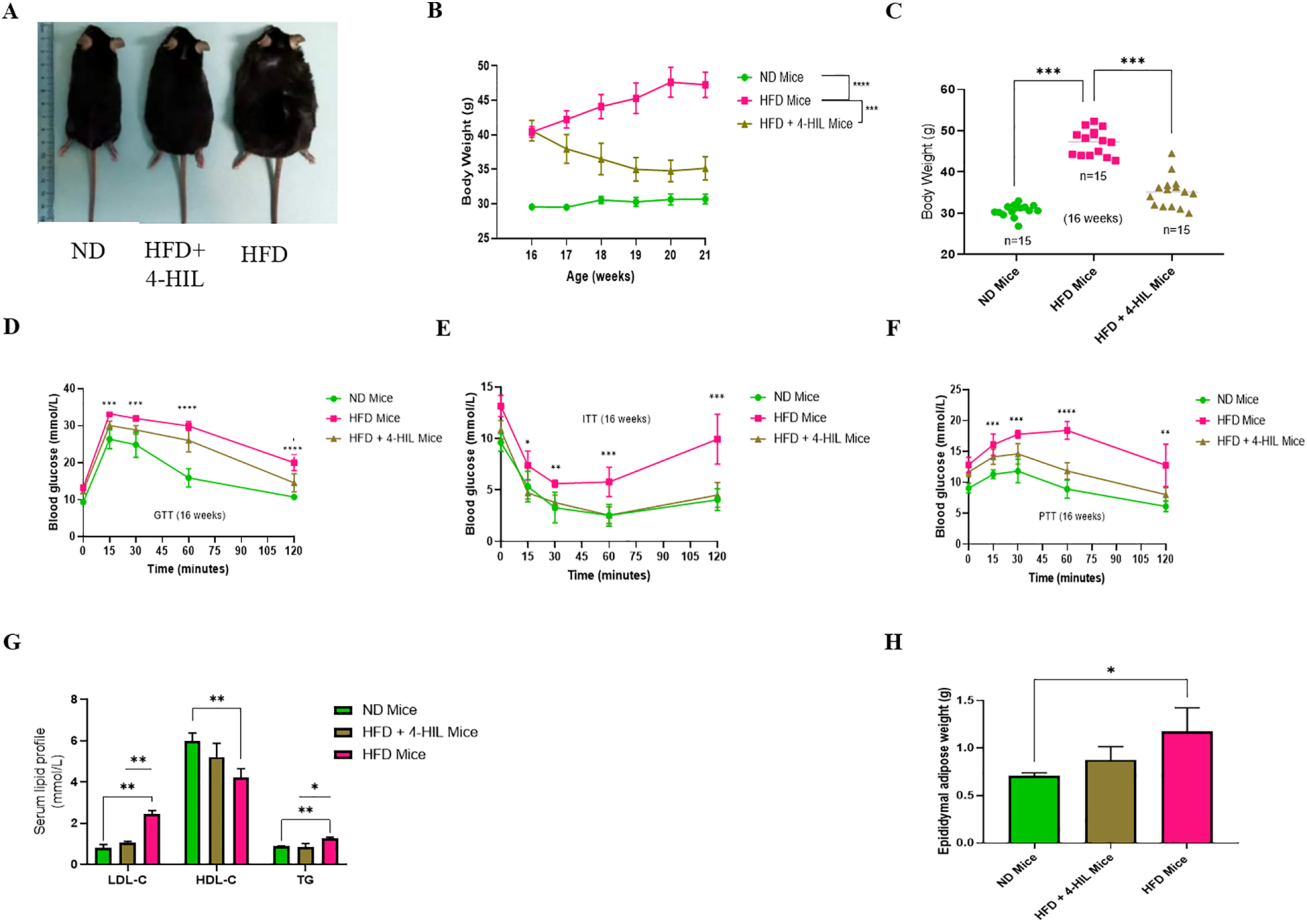
4-Hydroxyisoleucine (4-HIL) improves body weight, blood glucose concentration, serum lipid profiles, and epididymal adipose weight in male mice with obesity. **(A)** Representative images of the mice in each group after the 16th weeks of feeding and treatment. **(B, C)** Body weights of ND mice, HFD mice, and mice treated with 4-HIL, n=15 mice per group after 16 weeks of mouse modeling. Blood glucose levels during **(D)** GTT, **(E)** ITT, and **(F)** PTT after 16 weeks (n=5 mice per group). **(G)** LDL-c, HDL-c and TG concentrations in mice groups after 16 weeks, n=3 per group. **(H)** Epididymal adipose weight of mice after 16 weeks (n=3 per group). *P < 0.05; **P < 0.01; ***P < 0.001; ****P < 0.0001.

To determine the effect of 4-hydroxyisoleucine (4-HIL) on body weight reduction, we first developed mice with diet-induced obesity after ten weeks of high fat diet feeding (**Supplementary Figure S1A**). Subsequently, 4-hydroxyisoleucine was administered daily for 6 weeks. The body weights of mice fed a normal diet (ND), high-fat diet only (HFD), and high-fat diet treated with 4-hydroxyisoleucine (HFD+4-HIL) were measured weekly (**Figures 1A–C**). Consistent with previous reports, we observed that 4-HIL treatment significantly reduced the body weight of mice in the HFD group compared to HFD mice without treatment (19) (**Figures 1A–C**).

To confirm that obesity is linked to metabolic disorders such as glucose intolerance and insulin resistance, metabolic tolerance tests, including glucose tolerance test (GTT), insulin tolerance test (ITT), and pyruvate tolerance test (PTT), were conducted. Prior to treatment, HFD mice (mice with obesity) had a significantly higher blood glucose concentration than ND mice (**Supplementary Figures S1B–D**). After six weeks of treatment, mice with obesity treated with 4-HIL showed improved glucose tolerance compared to HFD mice (**Figure 1D**). In addition, the insulin tolerance test (ITT) revealed that unlike mice with obesity that were highly resistant to insulin injection, mice treated with 4-HIL had a significantly lower blood glucose concentration, similar to that in ND mice (**Figure 1E**). The pyruvate tolerance test (PTT) results showed a significant enhancement in blood glucose concentration in 4-HIL treated mice compared to that in mice with obesity (**Figure 1F**).

Furthermore, before the mice with obesity were treated with 4-HIL, they exhibited high serum concentrations of low-density lipoprotein cholesterol (LDL-c) and triglycerides (TG) and low serum concentrations of high-density lipoprotein cholesterol (HDL-c) (**Supplementary Figure S1E**). However, after treatment, the same lipid parameters were found to be remarkably optimized in 4-HIL treated mice compared to those without treatment (**Figure 1G**). This indicates the possibility of using 4-HIL to reduce dyslipidemia caused by a high-fat diet. Lastly, we found reduced epididymal adipose weight in 4-HIL mice compared with that in mice with obesity (**Figure 1H**).

These observations suggest that excess accumulation of adipose tissue, insulin resistance, and dyslipidemia can be controlled by 4-hydoxyisoleucine treatment.

### 3.2 Fertility indicators were enhanced in 4-hydroxyisoleucine-treated mice

**Figure 2:**
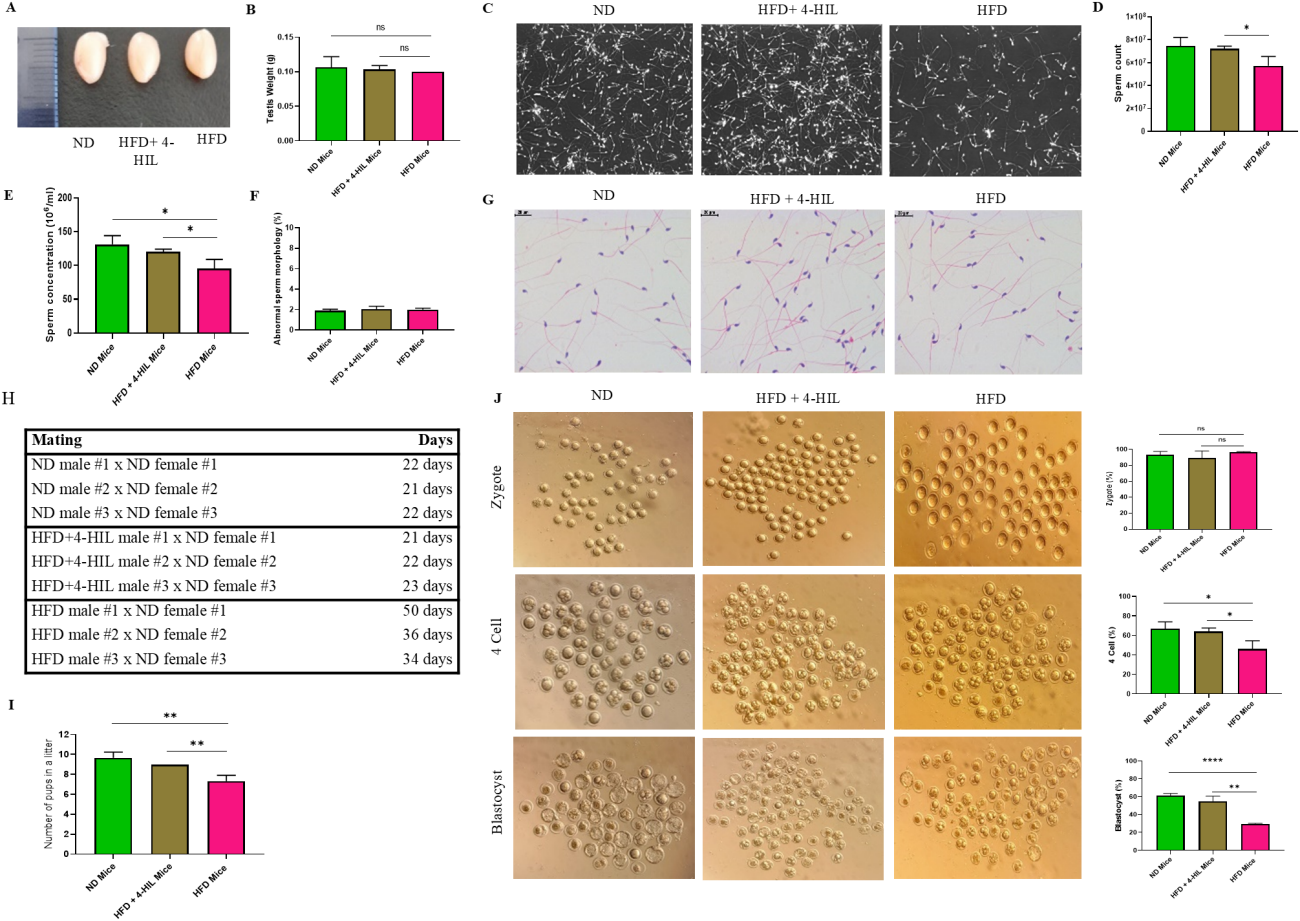
4-Hydroxyisoleucine enhances male fertility by increasing sperm count and concentration and improves the prognosis of IVF. **(A)** Images of testes in mice groups. **(B)** Testis weight in mice groups, n=3 per group. **(C-E)**. Epididymal sperm count and concentration in mouse groups (n = 3 mice per group). **(F, G)** Abnormal epididymal sperm morphology in mice (n = 3 mice per group). **(H, I)**. Number of pups born by mating ND, 4-HIL, and HFD males with C57BL/6 females (8 weeks old) to examine fertility, n=3 mice per group. **(J)** Zygotes, four-cell embryos, and blastocysts were generated from ND, HFD+ 4-HIL, and HFD mice groups, and 8-week-old C57BL/6 female mice (n=3 mice per group). Ns, not significant; *P < 0.05; **P < 0.01; ****P < 0.0001.

Male subfertility is a complication of obesity (11). Therefore, we explored the therapeutic effects of 4-HIL on fertility in male mice with diet-induced obesity. We first examined the testes of mice from all three (3) groups after 16 weeks of feeding and treatment and found no significant difference in testis weight among the groups (**Figures 2A, B**). Next, we collected epididymis from ND, HFD, and 4-HIL mice for computer-assisted sperm analysis (CASA). Based on the CASA results, 4-HIL treated mice had a significantly higher sperm count and concentration than those in the HFD group (**Figures 2C–E**). However, sperm morphology in all three mice groups was less defective, with no significant differences (**Figures 2F, G**). In addition, we randomly selected three male mice from each group and allowed them to mate with three C57BL/6 female mice of a normal background (8 weeks old), with one male mouse paired with one female mouse (1:1). We noticed that 4-HIL treated mice were able to impregnate females in a shorter period and produced a significantly higher number of pups per litter than HFD group males (**Figures 2H, I**).

Next, we aimed to ascertain the progression of preimplantation embryos using *in vitro* fertilization (IVF). We observed that the fertilization rates among the mice groups were high, with no significant difference in the number of zygotes produced (**Figure 2J**). However, the percentage of 4-cell embryos produced from the sperms of 4-HIL treated mice was significantly lower than that of 4-cell embryos from normal diet-fed mice. In addition, there was a significantly reduced number of 4-cell embryos obtained from mice with obesity (HFD group) compared to that of 4-HIL treated mice (**Figure 2J**). Approximately 30% of the embryos from mice in the HFD group progressed to the blastocyst stage, which was lower than the 55% and 61% of blastocysts derived from 4-HIL treated and normal control mice, respectively (**Figure 2J**). These results suggest that 4-hydroxyisoleucine treatment could enhance fertility of male mice with obesity.

### 3.3 4-HIL therapy reduces congestion of germinal epithelium in the seminiferous lumen, excessive lipid deposition in testicular tissue, and apoptosis of testicular cells

**Figure 3:**
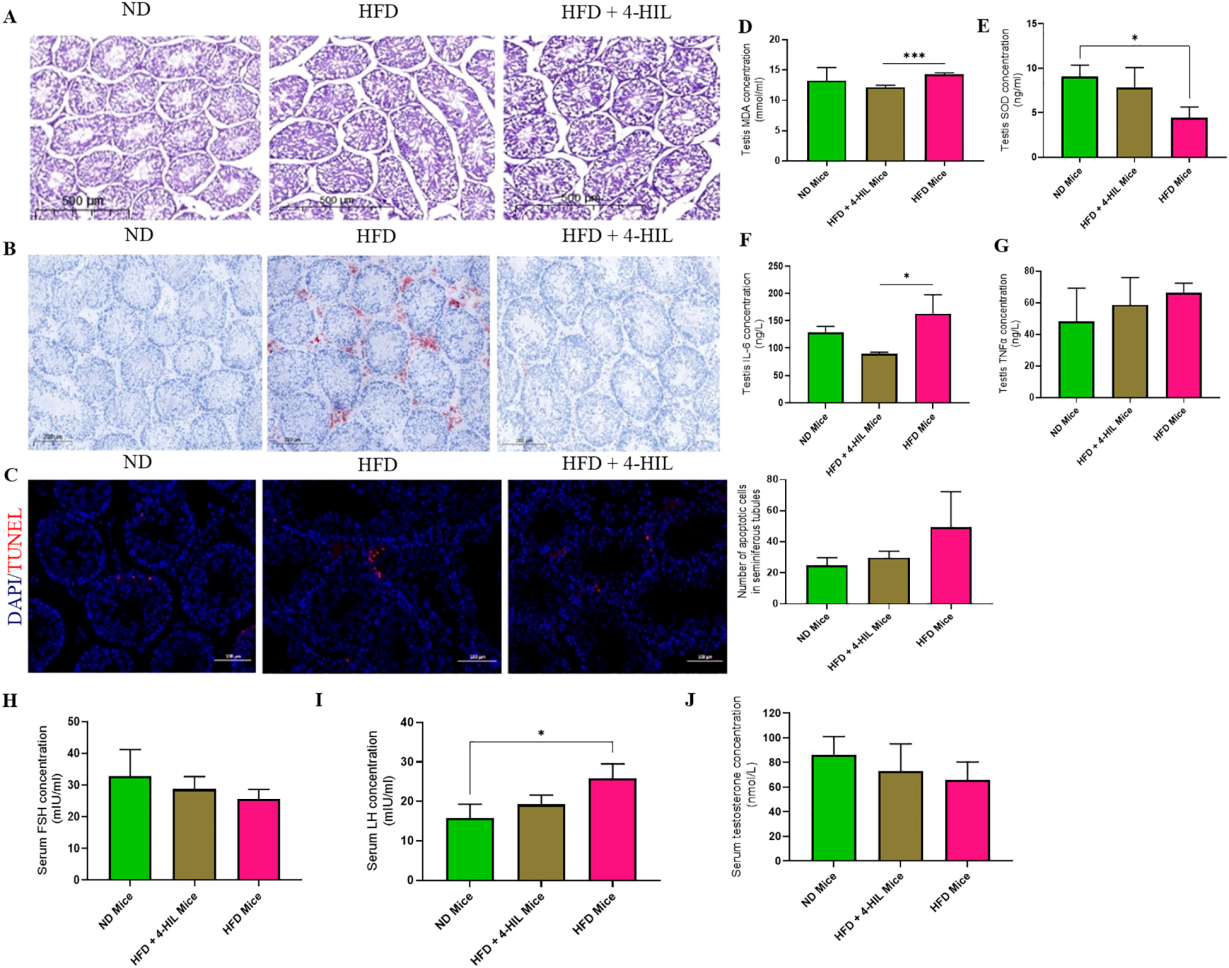
4-Hydroxyisoleucine reduces histopathologies, oxidative stress, and inflammation in the testis and enhances serum concentrations of spermatogenic hormones. **(A)** Representative images of H&E-stained testicular sections from mice. **(B)** Lipid deposition in testes of mice. **(C)** TUNEL-positive cells within seminiferous tubules in the testes of mice, n=3 per group. **(D)** Testis MDA **(E)** Testis SOD **(F)** Testis IL-6 and **(G)** Testis TNFα in mice groups, n=3 per group. **(H)** Serum FSH **(I)** Serum LH and **(J)** Serum testosterone in mice (n=3 per group). *P < 0.05; ***P < 0.001.

Although there were no obvious differences in testis weight among the three groups, it has been reported that high-fat diet consumption has an adverse effect on the male germinal epithelium, interstitium of the testis, and testicular cells (13). We hypothesized that 4-hydroxyisoleucine treatment would reduce these adverse effects. To address this, we conducted a histological assessment (hematoxylin and eosin staining) of testes from the three mice groups. Histological examination revealed irregularly arranged germinal epithelia, leading to congestion in the lumen of the seminiferous tubules of the testes from HFD group mice; however, 4-HIL treated mice had less crowded germinal epithelia, leading to visible boundaries in the lumen of the seminiferous tubules (**Figure 3A**). Additionally, Oil Red O staining revealed intense lipid deposition in the peritubular spaces and interstitium of the testes of HFD group mice, as opposed to the testes of 4-HIL treated mice (**Figure 3B**). Furthermore, in terminal deoxynucleotidyl transferase dUTP nick end labeling (TUNEL) assays, we observed a higher number of apoptotic cells in HFD group mice than in the 4-HIL treatment group; however, no statistically significant difference was established (**Figure 3C**). These results clearly indicate the ameliorative effects of 4-HIL on HFD-induced histopathological changes in the testes.

### 3.4 4-HIL treatment reduces oxidative stress and inflammation in testis and enhances concentrations of spermatogenic hormones in serum

A previous study reported high oxidative stress in the testes of mice with diet-induced obesity, as shown by the testicular concentrations of malondialdehyde (MDA) and superoxide dismutase (SOD) (13). Therefore, we assessed the levels of superoxide dismutase (SOD) and malondialdehyde (MDA) in the serum and testes of mice. We found a decrease and significant reduction of MDA concentration in the serum and testis respectively of 4-HIL-treated mice compared to those in the HFD group (**Supplementary Figure S2A**, **Figure 3D**). Similarly, in 4-HIL-treated mice, SOD levels were optimized, with a restoration of SOD concentration in the serum and testis (**Supplementary Figure S2B**, **Figure 3E**). In addition, inflammatory markers, including interleukin-6 (IL-6) and tumor necrosis factor-alpha (TNFα), were assessed, and their serum and testis concentrations were found to be reduced in 4-HIL treated mice compared to those in HFD mice (**Supplementary Figures S2C, D**; **Figures 3F, G**). 4-HIL treatment improved spermatogenic hormone levels, but no statistically significant differences were observed. FSH levels were higher in 4-HIL-treated mice than in HFD mice (**Figure 3H**), and LH levels within 4-HIL mice were similar to those of ND mice (**Figure 3I**), and testosterone levels were lower in HFD mice than in 4-HIL-treated mice (**Figure 3J**).

### 3.5 Genes involved in spermatogenesis are enhanced in the testis of 4-HIL-treated mice

**Figure 4:**
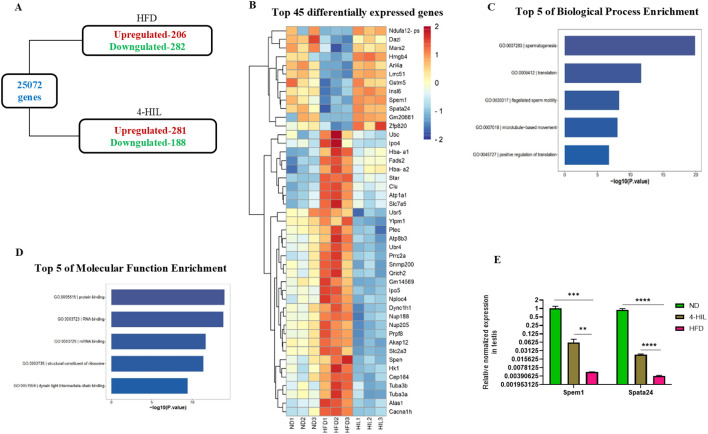
4-Hydroxyisoleucine upregulates spermatogenic gene expression. **(A)** The total number of expressed genes and differentially expressed genes in mice with obesity (HFD) and 4-HIL treated mice (4-HIL) were quantified by mRNA sequencing. **(B)** Heatmap, demonstrating top 45 significant differentially expressed genes from mRNA sequencing of testes of mice fed with normal diet (ND), mice with obesity (HFD), and 4-HIL treated mice (4-HIL). **(C)** Top five biological process enrichment from mRNA sequencing of testes in mice groups. **(D)** Top 5 of molecular function enrichment from mRNA sequencing of testes in mice groups. **(E)** RT-qPCR shows the relative normalized expression of *Spem1* and *Spata24* in testes from mice. **P < 0.01; ***P < 0.001, and ****P < 0.0001.

To determine the molecular mechanism by which 4-HIL enhances fertility in male mice with diet-induced obesity, we conducted mRNA sequencing of the testes of ND, obese (HFD), and 4-HIL mice. A total of 25,702 genes were identified (**Figure 4A**). Among these genes, 206 were upregulated and 282 were downregulated in the testes of the mice with obesity (**Figure 4A**). In addition, 281 genes were upregulated and 188 genes were downregulated in the testes of 4-HIL mice (**Figure 4A**). The top 45 significantly differentially expressed genes and gene ontology revealed spermatogenesis as a highly ranked biological process enrichment with *dazl*, *insl6*, *spem1*, and *spata24* upregulated in the testes of mice treated with 4-HIL and downregulated in the testes of mice with obesity (**Supplementary Figure S3A**; **Figures 4B, C**). In addition, the top-rated cellular component and molecular function were the cytoplasm and protein binding, respectively (**Supplementary Figure S3B**; **Figure 4D**). We validated the expressions of *spem1* and *spata24* using qPCR. The qPCR results revealed significantly higher relative normalized expression levels of *spem1* and *spata24* in the testes of mice treated with 4-HIL than in HFD group (**Figure 4E**).

## 4 Discussion

Obesity caused by unhealthy dietary habits is associated with metabolic dysfunctions, such as insulin resistance, dyslipidemia, and chronic inflammation, which can potentially disrupt testicular function and compromise fertility in men (12, 27). In this study, 4-HIL therapy in mice with diet-induced obesity displayed positive effects on metabolic parameters, which is consistent with the previously reported insulinotropic effects of 4-HIL (improved glucose tolerance and insulin sensitivity) (28). Additionally, the reduction in serum triglyceride and cholesterol levels highlights its lipid-lowering properties. These systemic benefits are likely to reduce metabolic stressors that may impair testicular function (29).

Oxidative stress and inflammation are key contributors to testicular damage in diet-induced obesity (30, 31). Increased levels of malondialdehyde (MDA) and reduced levels of superoxide dismutase (SOD) in the testes of mice with diet-induced obesity (13, 32), was observed in the HFD group in this study (an indication of high oxidative stress in the testes of mice with obesity), the 4-HIL treatment group showed a significant reduction in MDA levels and optimally restored SOD levels in the testes, indicating an antioxidative effect of 4-HIL. Similarly, there was a reduction in cytokine inflammatory markers, including interleukin-6 (IL-6) and tumor necrosis factor-alpha (TNF-α), which further exemplify 4-HIL’s anti-inflammatory properties. These findings align with those of previous studies, suggesting that the mitigation of oxidative stress and reduction in inflammation can improve testicular function and spermatogenesis (33, 34).

Histological investigations revealed improvements in the testes of 4-HIL-treated mice, indicating direct protective effects on testicular tissue. There was substantial lipid deposition, congestion of the seminiferous tubules, and increased apoptosis in the testes of mice in the HFD group, which were markedly reduced following 4-HIL treatment. These findings are consistent with those of previous studies demonstrating that lipid accumulation and apoptosis in the testes potentially disrupt spermatogenesis (17, 35). 4-HIL reversed these pathological changes and appeared to preserve the structural integrity of the testes, thereby supporting normal spermatogenesis. Furthermore, the hypothalamic-pituitary-gonadal (HPG) axis also plays a critical role in spermatogenesis regulation, and in the state of obesity, there is HPG disruption leading to hormonal imbalances including FSH, LH, and testosterone (17). Following treatment with 4-HIL, the study observed an improvement in testosterone levels and increased FSH concentrations, suggesting that 4-HIL helps optimize the endocrine environment required for spermatogenesis. These hormonal changes may have contributed to the observed improvements in sperm production and concentration.

The upregulation of spem1 and spata24 in the testes of 4-HIL-treated mice provides novel insights into the molecular mechanisms underlying the fertility-enhancing effects. These genes are critical regulators of spermatogenesis, and their downregulation in mice with obesity highlights molecular disruptions caused by obesity (36, 37). By restoring their expression, 4-HIL may directly influence the gene networks involved in sperm development and maturation in mice. Although the precise mechanism remains to be elucidated, our findings highlight a promising area for further research. This study had certain limitations; thus, the effects of 4-HIL were evaluated using a relatively small sample size and short treatment period. Therefore, long-term studies are required to assess the sustainability of the observed benefits. Key genes have been identified; however, the downstream pathways and precise molecular interactions remain unclear and warrant further investigation. Despite these limitations, the strength of this study lies in the integration of metabolic, histological, hormonal, and molecular analyses to provide a holistic understanding of the effects of 4-HIL on male fertility. The first study to describe key gene modulation by 4-HIL paves the way for further molecular studies. Moreover, the ability of 4-HIL to simultaneously improve systemic metabolic health and testicular function makes it a multifaceted intervention.

In conclusion, this study provides compelling evidence for the potential application of 4-HIL as a novel therapeutic agent for the treatment of obesity-induced male subfertility. The 4-HIL, by targeting both systemic metabolic dysfunction and testicular-specific impairments, offers a dual-action approach that could be advantageous for use in clinical settings. However, the translational applicability of 4-HIL requires further validation in human trials and long-term studies to ensure its safety and efficacy. For future perspective, further studies on the exploration of molecular pathways influenced by 4-HIL, particularly its role in regulating spermatogenesis-related genes, could provide deeper insights into its mechanisms of action. With the increasing prevalence of obesity and its associated reproductive challenges, interventions such as 4-HIL holds significant promise for improving fertility outcomes of men with obesity. Additionally, investigation into its potential epigenetic effects would be valuable, as obesity-induced epigenetic alterations may contribute to transgenerational reproductive consequences.

## Data Availability

The datasets presented in this study can be found in online repositories. The names of the repository/repositories and accession number(s) can be found in the article/**Supplementary Material**.
