# Supplementary material for: Potential role of 4-hydroxyisoleucine in enhancing fertility in male mice with diet-induced obesity
Source: Front Endocrinol (Lausanne). 2025 Aug 21;16:1561543. doi: 10.3389/fendo.2025.1561543 (PMC12408301; doi:10.3389/fendo.2025.1561543)
Supplement: Supplementary file 1 [file DataSheet1.docx]

**Supplementary Material**

**Potential Role of 4-Hydroxyisoleucine in Enhancing Fertility in Male Mice with Diet-Induced Obesity**

**Emmanuel Osei Nkansah^1^, Yunzhu Lan^1^, Hui Zhang^1^, Binbin Xu^1^, Qiaodan Li^1^, Mohammad Ishraq Zafar^1*^, Jian Xu^1*^.**

^1^Department of Obstetrics and Gynecology, Center for Reproductive Medicine, The Fourth Affiliated Hospital of School of Medicine and International School of Medicine, International Institutes of Medicine, Zhejiang University, Yiwu, Zhejiang, China

***Correspondence**

**Corresponding Author:** Mohammad Ishraq Zafar

Email: [zafar@zju.edu.cn](mailto:zafar@zju.edu.cn)

**Corresponding Author:** Jian Xu

Email: [xuj@zju.edu.cn](mailto:xuj@zju.edu.cn)


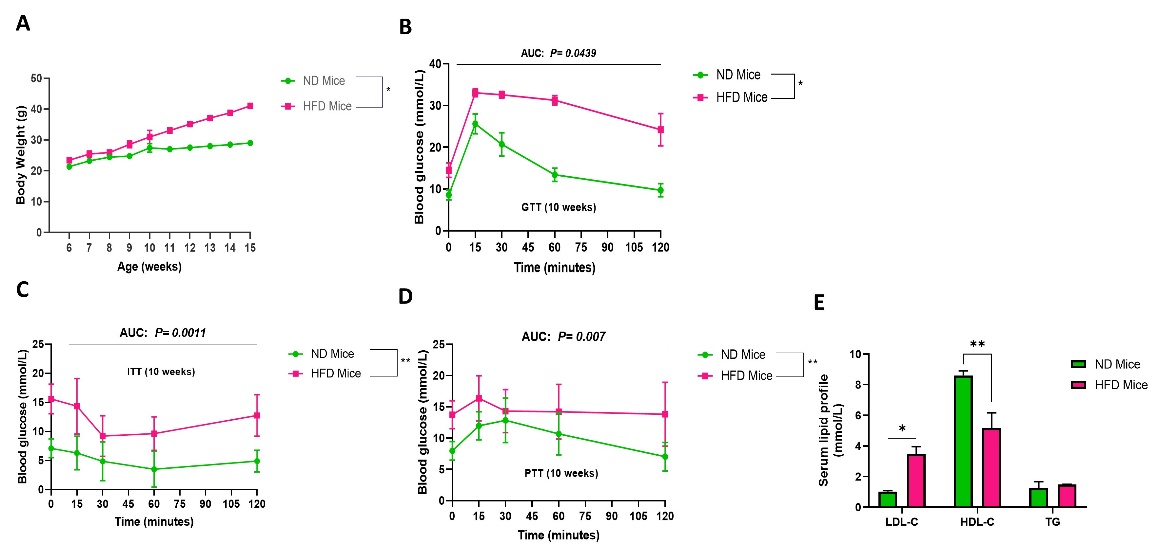


**Figure S1, related to Figure 1. 4-Hydroxyisoleucine (4-HIL) improves body weight, blood glucose concentration, serum lipid profiles, and epididymal adipose weight in male mice with obesity.**

**A.**  Body weight of ND and HFD mice before 4-HIL treatment, n=15 for each group. **B.** Glucose tolerance test before 4-HIL treatment. **C.** Insulin tolerance test before 4-HIL treatment. **D.** Pyruvate tolerance test before 4-HIL treatment. n=5 mice per group**. E.** Low-density lipoprotein cholesterol (LDL-c), High-density lipoprotein cholesterol (HDL-c), and triglyceride (TG) concentrations in mice groups before 4-HIL treatment, n=3 per group. *****P < 0.05; ******P < 0.01.


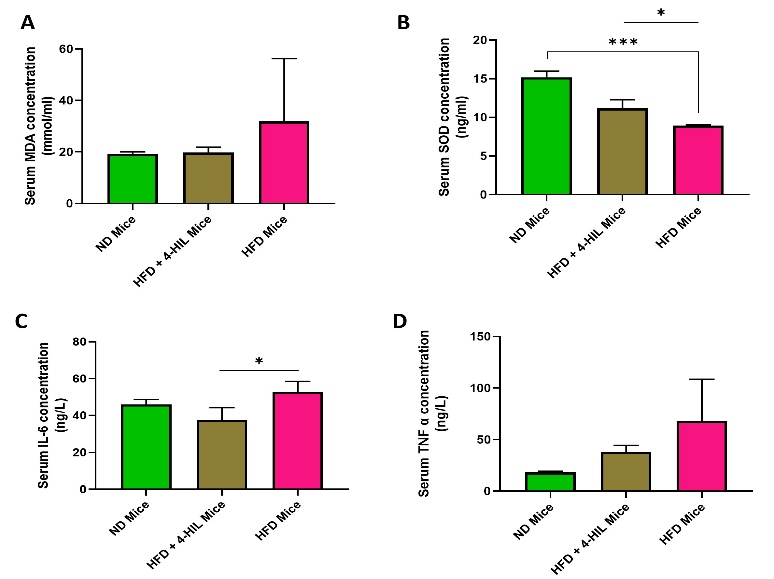


**Figure S2, related to Figure 3. 4-Hydroxyisoleucine reduces histopathologies, oxidative stress, and inflammation in the testis and enhances serum concentrations of spermatogenic hormones**

**A.** Serum MDA **B.** Serum SOD **C.** Serum IL-6 and **D.** Serum TNFα in mice groups, n=3 per group. *****P < 0.05; *******P < 0.001.


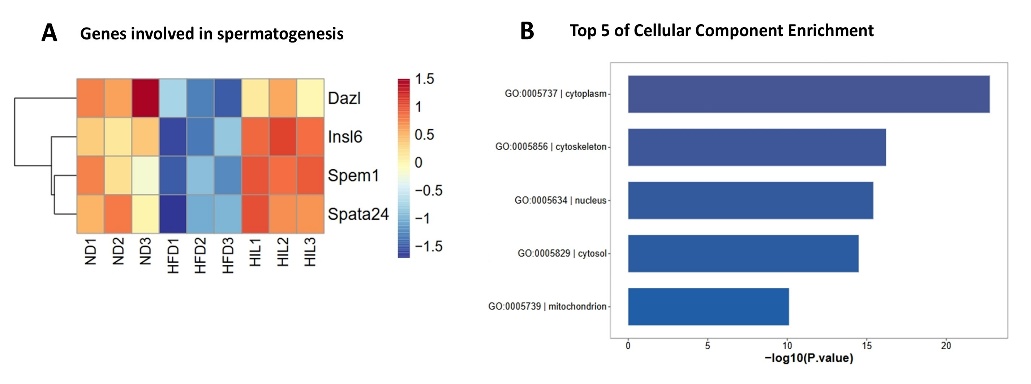


**Figure S3, related to Figure 4. 4-Hydroxyisoleucine upregulates spermatogenic gene expression.**

**A**. Heatmap, demonstrating differential expression of spermatogenic genes (*dazl*, *Insl6*, *spem1*, and *spata24*) from mRNA sequencing of testes of mice fed with normal diet (ND), mice with obesity (HFD), and 4-HIL treated mice (4-HIL), n=3 per group. **B.** Top 5 of cellular component enrichment from mRNA sequencing of testes in mice groups, n=3 per mice group.

The accession number for mRNA sequencing files is PRJNA1195381 and its web link is http://www.ncbi.nlm.nih.gov/bioproject/1195381
